# A Remote Patient-Monitoring System for Intensive Care Medicine: Mixed Methods Human-Centered Design and Usability Evaluation

**DOI:** 10.2196/30655

**Published:** 2022-03-11

**Authors:** Akira-Sebastian Poncette, Lina Katharina Mosch, Lars Stablo, Claudia Spies, Monique Schieler, Steffen Weber-Carstens, Markus A Feufel, Felix Balzer

**Affiliations:** 1 Institute of Medical Informatics Charité – Universitätsmedizin Berlin Corporate Member of Freie Universität Berlin and Humboldt-Universität zu Berlin Berlin Germany; 2 Department of Anesthesiology and Intensive Care Medicine Charité – Universitätsmedizin Berlin, Corporate Member of Freie Universität Berlin and Humboldt-Universität zu Berlin Berlin Germany; 3 Division of Ergonomics Department of Psychology and Ergonomics (IPA) Technische Universität Berlin Berlin Germany

**Keywords:** digital health, patient monitoring, intensive care medicine, intensive care unit, technological innovation, user-centered design, usability, user experience, implementation science, qualitative research, interview, mixed methods, mobile phone

## Abstract

**Background:**

Continuous monitoring of vital signs is critical for ensuring patient safety in intensive care units (ICUs) and is becoming increasingly relevant in general wards. The effectiveness of health information technologies such as patient-monitoring systems is highly determined by usability, the lack of which can ultimately compromise patient safety. Usability problems can be identified and prevented by involving users (ie, clinicians).

**Objective:**

In this study, we aim to apply a human-centered design approach to evaluate the usability of a remote patient-monitoring system user interface (UI) in the ICU context and conceptualize and evaluate design changes.

**Methods:**

Following institutional review board approval (EA1/031/18), a formative evaluation of the monitoring UI was performed. Simulated use tests with think-aloud protocols were conducted with ICU staff (n=5), and the resulting qualitative data were analyzed using a deductive analytic approach. On the basis of the identified usability problems, we conceptualized informed design changes and applied them to develop an improved prototype of the monitoring UI. Comparing the UIs, we evaluated perceived usability using the System Usability Scale, performance efficiency with the normative path deviation, and effectiveness by measuring the task completion rate (n=5). Measures were tested for statistical significance using a 2-sample *t* test, Poisson regression with a generalized linear mixed-effects model, and the N-1 chi-square test. *P*<.05 were considered significant.

**Results:**

We found 37 individual usability problems specific to monitoring UI, which could be assigned to six subcodes: usefulness of the system, response time, responsiveness, meaning of labels, function of UI elements, and navigation. Among user ideas and requirements for the UI were high usability, customizability, and the provision of audible alarm notifications. Changes in graphics and design were proposed to allow for better navigation, information retrieval, and spatial orientation. The UI was revised by creating a prototype with a more responsive design and changes regarding labeling and UI elements. Statistical analysis showed that perceived usability improved significantly (System Usability Scale design A: mean 68.5, SD 11.26, n=5; design B: mean 89, SD 4.87, n=5; *P*=.003), as did performance efficiency (normative path deviation design A: mean 8.8, SD 5.26, n=5; design B: mean 3.2, SD 3.03, n=5; *P*=.001), and effectiveness (design A: 18 trials, failed 7, 39% times, passed 11, 61% times; design B: 20 trials, failed 0 times, passed 20 times; *P*=.002).

**Conclusions:**

Usability testing with think-aloud protocols led to a patient-monitoring UI with significantly improved usability, performance, and effectiveness. In the ICU work environment, difficult-to-use technology may result in detrimental outcomes for staff and patients. Technical devices should be designed to support efficient and effective work processes. Our results suggest that this can be achieved by applying basic human-centered design methods and principles.

**Trial Registration:**

ClinicalTrials.gov NCT03514173; https://clinicaltrials.gov/ct2/show/NCT03514173

## Introduction

### Background

Continuous monitoring of vital signs is essential for patient safety in the intensive care unit (ICU) and emergency room [1]. It is also becoming increasingly relevant in general wards [2]. In the past decade, particularly in the context of the digital transformation of health care, vital sign monitoring has undergone constant change and is being transformed and augmented by important technological innovations such as less invasive sensors, remote monitoring technology [3-5], and artificial intelligence for clinical decision support [6,7]. Together, these innovations hold great promise for improving patient safety and health care provision [8,9].

Effective implementation of novel technologies, such as remote patient-monitoring devices, faces a variety of barriers [10-12], including lack of adoption by clinicians, often because of poor usability of the respective technologies [13-15]. In addition to its importance in successful implementation, usability is closely related to the efficacy of the technology [16,17]. A lack of usability may lead to medical errors, thus compromising patient safety [18,19]. Therefore, usability evaluation and identification of specific usability problems are essential in the development of a novel technology and its implementation in the clinical setting. However, to date, usability problems remain prominent in health information technology (IT), suggesting that usability aspects are often neglected in the health IT development process [20-22].

The human-centered design (HCD) approach is centered on the involvement of end users and their experiences with the product throughout the design and development process [23]. Applying HCD in the early stages of the design of novel digital health technologies can improve usability, staff adoption, effectiveness, and efficiency [24,25]. Several frameworks and guidelines for redesigning health care interfaces in accordance with HCD have been published; however, their adoption in health care has been lagging, and evidence on the impact of this topic on clinical performance outcomes is scarce [26-32].

### Aim

We aim to evaluate the usability of a remote patient-monitoring system and, specifically, identify usability problems, positive findings, and user ideas. We hypothesize that an HCD approach will help to implement evidence-based design changes that will improve the subjectively perceived usability and objective measures of the effectiveness and efficiency of the technology.

## Methods

### Ethics Approval and Consent to Participate

This study was approved by the ethics committee of the Charité–Universitätsmedizin Berlin (EA1/031/18). All participants provided consent before the study.

### Study Design

**Figure 1:**
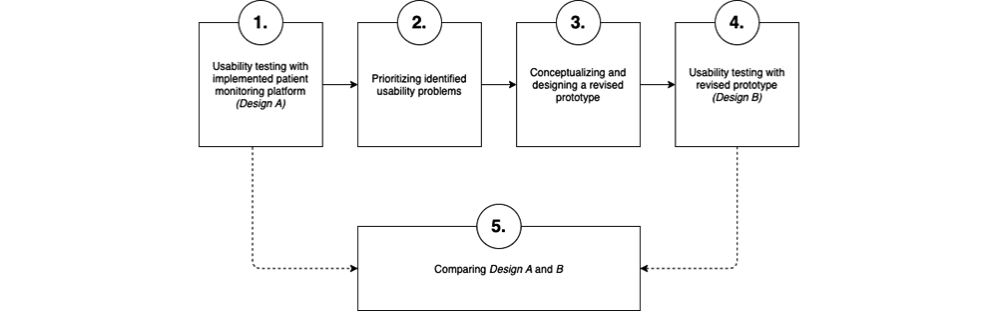
The research approach, beginning with usability testing and identification of major problems in design A, followed by prototyping of design B and its usability testing, concluding with a comparison between design A and design B.

Our usability study followed a five-step, mixed methods approach (Figure 1): (1) formative usability test of the implemented patient-monitoring platform interface design A [33], (2) identification and prioritization of usability problems, (3) conceptualization and design of prototype interface design B with informed design changes, (4) formative usability testing of design B, and (5) comparison of design A and design B. For usability testing, we applied simulated use tests with think-aloud protocols and performance measurements (subjectively perceived usability, efficiency, and effectiveness) [30,34]. For step 5, we chose a single-factor 2-group study design, as described by Gravetter and Forzano [35].

### Study Setting and Technical Setup

This study was conducted in the context of implementing the Vital Sync 2.4 virtual patient-monitoring platform (Medtronic plc) in the Post Anesthesia Care Unit, an ICU primarily for postoperative patients requiring short-term intensive care treatment and monitoring. VitalSync was used to monitor patients in the ICU from portable tablet computers on hospital premises. The primary patient-monitoring system used was the IntelliVue patient monitoring system (MX800 software, version M.00.03; MMS X2 software, version H.15.41-M.00.04) from Koninklijke Philips NV.

**Figure 2:**
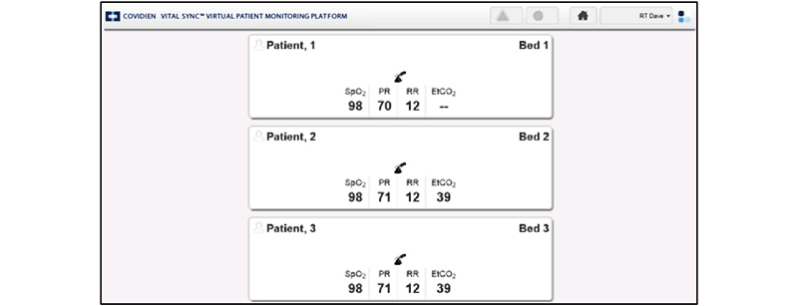
Home screen of the implemented patient-monitoring platform (design A). etCO2: end-tidal carbon dioxide; PR: pulse rate; RR: respiratory rate; SPO2: peripheral capillary oxygen saturation [36-38].

**Figure 3:**
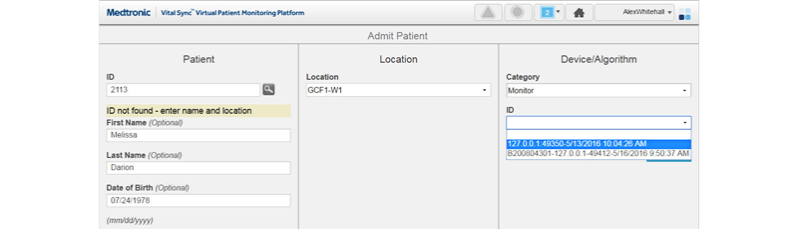
Admit Patient screen of the implemented patient-monitoring platform (design A) [36-38].

Between May 2018 and June 2019, the VitalSync monitoring system was installed for 5 of the 10 ICU beds. Two sensors (for pulse oximetry and capnography) recorded peripheral capillary oxygen saturation, pulse rate, end-tidal carbon dioxide, and respiratory rate at a frequency of 1 Hz. The VitalSync user interface (UI) was displayed on a monitor at the central station and on six tablet computers (2 standard iPads, 2 iPad minis, and 2 Microsoft Surfaces). The UI of the system was structured where the home screen gave an overview of patients admitted to the system, displayed in tiles (Figure 2). Displayed were numerical values for the monitoring parameters, the patient’s name and bed location, and specific information on alarms if any. Clicking on a patient tile took the user to a screen with details about the selected patient (eg, graphical curves for end-tidal carbon dioxide values) and other functions (eg, displaying patient reports, linking, or unlinking devices). There was also the option of clicking on each parameter to see a trend analysis of that value. To link a patient to the system, the *Admit Patient* screen was accessed, and the patient ID was entered, after which the bed location and monitoring device could be selected to complete the admission process (Figure 3) [36-38]. Further technical description and details regarding the use of the software can be found elsewhere [10].

### Research Team

Following the principles of HCD [39], our research team members have multidisciplinary skills and perspectives. Specifically, the team included a physician with a background in anesthesiology, intensive care medicine, geriatrics, and digital health (ASP); a senior medical student with a focus on digital health (LM); a senior human factors student with a background in engineering (LS); a professor of ergonomics with a PhD in human factors and industrial and organizational psychology (MF); the anesthesiology department’s head of staff (CS); and a professor of medical data science, who is also a consultant anesthesiologist and computer scientist (FB).

### Data Collection

Data collection took place from August 23, 2019, to March 10, 2020. Our data comprised think-aloud transcripts of the first block of usability tests (ie, design A), researcher notes (including click patterns), and posttest questionnaires from the two blocks of usability tests (ie, design A and design B). We conducted 10 usability tests with ICU staff—5 (50%) tests each for design A (August and November 2019) and design B (February and March 2020). For recruitment, we contacted potential participants via email. We aimed to represent all professions working with the remote patient-monitoring system, namely anesthesiologists (3/10, 30%), ICU nurses (5/10, 50%), and respiratory therapists (2/10, 20%). Participation was voluntary, and no incentives were offered.

Usability testing of design A and design B was performed on an iPad mini 4 (model A1550). For testing sessions with design A, 5 patients in the ICU were connected to the system. This allowed real-time monitoring of the patients’ vital signs on the iPad used by the participants. Testing of design B differed from testing of design A in that no patients were connected to actual sensors, and only one of the researchers was present during the testing sessions.

The testing sessions were conducted in German. Participants were asked about their profession and the number of years of professional experience in intensive care medicine. They were then given 4 tasks to complete while verbalizing their thoughts [40]. We provided the participants with the following use context: “A new patient was admitted to the unit and was connected to the etCO_2_ and SpO_2_ sensors (Mrs. Schmitt, born 01/01/1950, Patient-ID 12345, bed site 02)*.*”

In accordance with the requirements for formative usability testing [41], participants were selected to complete the following key tasks during the simulated use test:

1. “Please add Mrs. Schmitt to the patients you want to monitor in Vital Sync™.”
2. “You would like to see the trend of Mrs. Schmitt’s oxygen saturation for the last two hours. How do you proceed?”
3. “You have identified that Mrs. Schmitt is actually not in bed 2 but in bed 6. You want to adjust this information in Vital Sync™. How do you proceed?”
4. “Mrs. Schmitt has been discharged. Please disconnect Mrs. Schmitt’s devices and delete her entry from Vital Sync™.”

Audio recordings of the simulated use tests were transcribed verbatim. A researcher who had not performed the transcription reviewed the transcripts. Immediately after the simulated use tests of both designs A and B, participants were asked to complete a posttest questionnaire, including the System Usability Scale (SUS) [42,43].

### Data Analysis

#### Qualitative Analysis and Identification of Usability Problems

To analyze data from the think-aloud transcripts of design A testing sessions, we adapted a deductive analytic approach [44]. A coding scheme introduced by Kushniruk and Patel [44] was refined to the topic of study (patient monitoring in ICUs; Multimedia Appendix 1). Using the qualitative data analysis software MAXQDA 2018 (VERBI GmbH), think-aloud transcripts were coded according to the developed scheme. Coded segments (ie, usability problems) were specified into the subcodes, which were further summarized and listed (eg, meaning of labels unclear).

To decide which problems to eliminate first in the subsequent design iteration, summarized usability problems were ranked in terms of severity and frequency [45,46]. To assess problem severity, impact scores were assigned to each usability problem by 2 physicians who were experienced in intensive care medicine. The following scores were available for selection:

- The solution to this problem is subtle and possible enhancement or suggestion (score 1)
- The problem has a minor effect on usability (score 2)
- The problem creates significant delay and frustration (score 3)
- The problem prevents task completion (score 4)

Subsequently, the probability of occurrence was calculated by dividing the number of participants who encountered a particular problem by the total number of participants. To categorize problem frequency, each usability problem was assigned to one of four frequency levels: frequency ≤10% (level 1), frequency 11% to 50% (level 2), frequency 51% to 89% (level 3), and frequency ≥90% (level 4). Finally, criticality was calculated by adding the impact score and frequency levels [45] (eg, when a usability problem was rated as creating significant delays [impact score 3], which was experienced by 80% of participants [level 3], resulting in a criticality score of 6).

#### Analysis of Effectiveness, Efficiency, and Subjective Usability

The task completion rate [47,48] was measured to evaluate the effectiveness of design A and design B. Normative path deviation [49] was assessed based on participants’ click patterns to account for efficiency. The sequence of steps users took when interacting with the interface to complete a task was compared with an optimal sequence of goal-directed steps defined by the researchers for each task. The difference between the normative path and observed path for each user and each task was calculated using the Levenshtein algorithm [33,49]. The SUS was used to assess the perceived usability of design A and design B [42,43,50].

#### Prototype Design

Design solutions were conceptualized by ASP and LS for all identified usability problems. This resulted in a list of ranked usability problems with the suggested design solutions. The identified usability problems from design A were revised by building design B, a clickable prototype, using Axure RP 9. A feedback loop was used to develop design B: one researcher (LS) built the prototype, and another researcher (ASP) reviewed the design and provided feedback from an intensivist’s perspective.

#### Statistical Analysis

To assess the level of improvement between design A and design B, we hypothesized that the task completion rate for design B would be higher than that of design A, design B would lead to lower normative path deviation values than design A, and the SUS scores for design B would be higher than that of design A.

We used the N-1 chi-square test to compare the task completion rates of both designs [45]. To compare the normative path deviations for both designs, we used a Poisson regression drawing upon a generalized linear mixed-effects model with participants as random effects, as introduced by Schmettow et al [33]. A 2-sample *t* test was conducted to compare the SUS scores between design A and design B, as recommended by Sauro and Lewis [45]. We tested for normality using the Shapiro–Wilk test [51] and homoscedasticity (homogeneity of variance) using the Levene test [52].

## Results

### Overview and Sample

Measured by task completion rate, normative path deviation, and SUS score, design B was found to be significantly improved compared with design A. We first elaborate on the results of the qualitative analyses and then report the quantitative results.

The sample comprised a total of 10 ICU staff, aged 25 to 39 years, with work experience ranging from 1 to 20 years, who were divided into groups (5, 50% each) for the evaluation of the 2 designs.

### Qualitative Results

#### Summary

**Figure 4:**
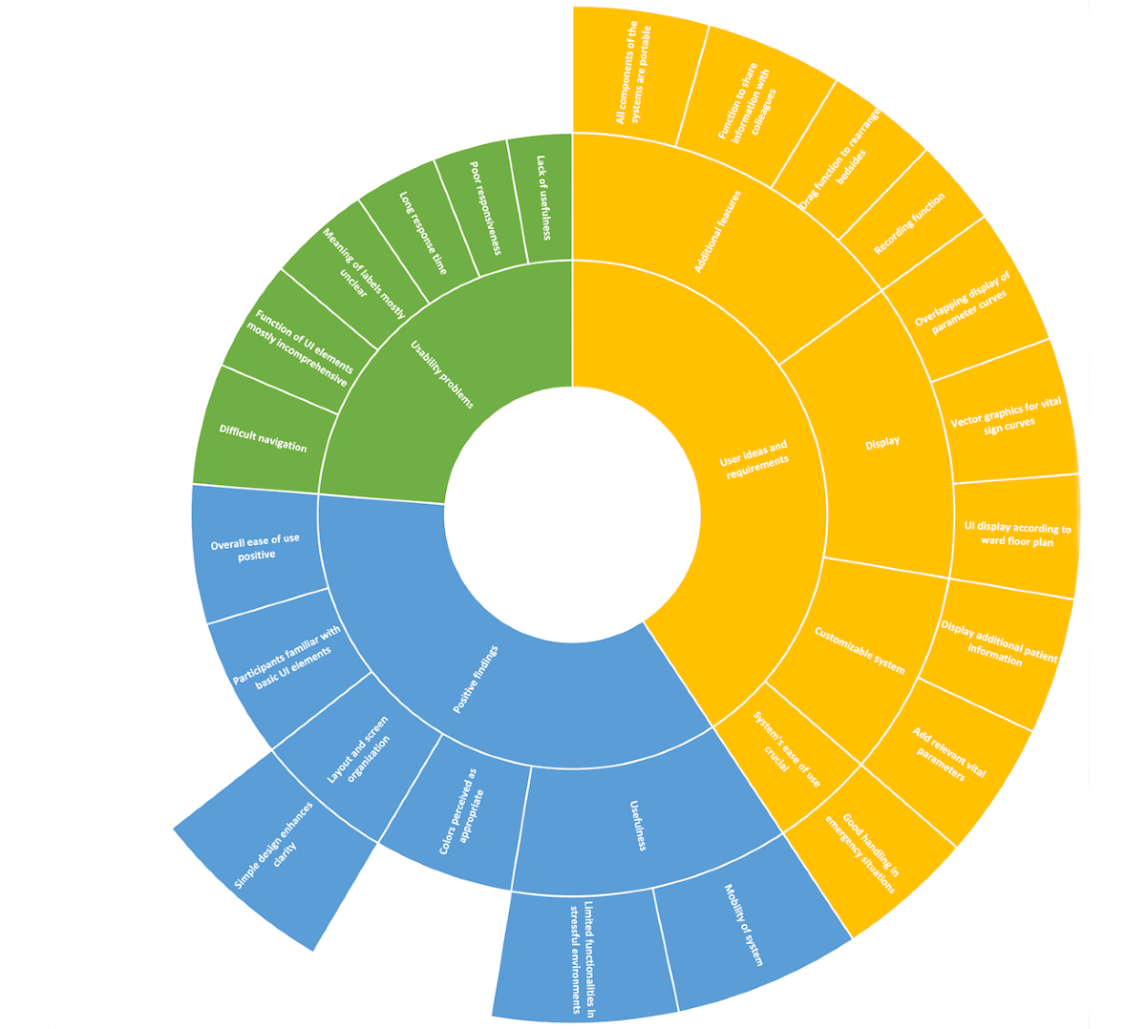
Results of qualitative analysis of the think-aloud transcript. Three main codes were identified (inner ring) and subcoded (middle ring). The outer ring represents further information derived from the concrete items that were assigned to the subcodes (ie, specific user ideas or positive findings). UI: user interface.

The coding of the transcripts revealed three main codes: usability problems, user ideas and requirements, and positive findings. The codes are visualized with a sunburst diagram (Figure 4; see Multimedia Appendix 1 for the adapted coding scheme by Kushniruk and Patel [44]). Items from the transcripts of the think-aloud protocols were mapped to the subcodes derived by Kushniruk and Patel [44] for the main categories—usability problems and positive findings. For usability problems, the items were assigned to the subcodes of usefulness of the system, response time, responsiveness, meaning of labels, function of UI elements, and navigation; for positive findings, the items were assigned to usefulness, overall ease of use, function of UI elements, layout/screen organization, and color.

#### Usability Problems

**Figure 5:**
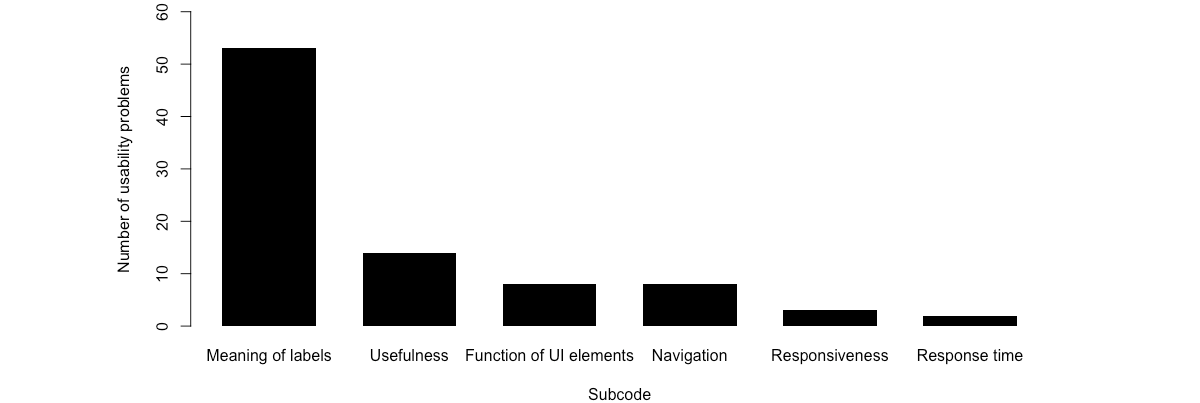
Number of occurrences for each subcode of usability problems. Meaning of labels (n=53), usefulness (n=14), function of UI elements (n=8), navigation (n=8), responsiveness (n=3), response time (n=2). UI: user interface.

In total, 37 specific usability problems were identified (Multimedia Appendix 2). The number of usability problems related to the respective codes is visualized in Figure 5; most issues were related to labeling (53/88, 60%). The meaning of labels was mostly unclear—that is, participants were not familiar with certain terms (eg, the meaning of exclamation marks, abbreviations such as those for pulse rate [PR] and integrated pulmonary index [IPI], or terms such as *polardiagramm*). Users were concerned about whether a certain function was useful for the requirements of their clinical work or when a given task could not be accomplished (eg, participants selected the wrong bed site tile and participants were not sure about the correct patient or device ID; 14/88, 16%). There were difficulties in using or understanding the function of UI elements such as buttons (eg, gray circle or telescope symbols; 8/88, 9%). Furthermore, participants seemed to have problems navigating the monitoring system (ie, finding the right click path to admit patients to the platform; 8/88, 9%). Users criticized the responsiveness of the system (ie, the system did not behave as expected; 3/88, 3%) and the response time (ie, they complained about the time it took the device to respond; 2/88, 2%).

#### User Ideas and Requirements

Users emphasized that the system’s ease of use was particularly important to ensure its usability in emergency situations. The tool should be customizable to add other relevant vital signs (eg, intracranial pressure) or to display additional patient information. Participants required audible alarm notifications and the ability to share information regarding relevant patient events with colleagues (eg, about critical patient conditions). Vector graphics were suggested to allow zooming in and out of the vital sign curves. Moreover, participants demanded the ability to see curves of different parameters in an overlapping representation to be able to make inferences from one vital parameter to another. To facilitate spatial orientation, it was suggested that the beds be displayed in the UI according to the physical ward floor plan. Other ideas included adding a drag-and-drop function to rearrange multiple beds at once in the UI and integrating a high-frequency recording function to capture critical events.

#### Positive Findings

Participants stated that the system’s scope of functionality was limited compared with other monitoring solutions. However, the reduced complexity was considered helpful in hospital wards with high patient turnover or stressful environments to get a quick overview of the patient’s health condition. The system’s mobility and overall ease of use were perceived as positive. Participants seemed to be familiar with the following basic UI elements: the home button depicted by a house, the editing symbol depicted by a pen, and the alarm symbol depicted by a warning triangle. Simplicity in the design and use of color was also rated as positive.

### Design Iteration

**Figure 6:**
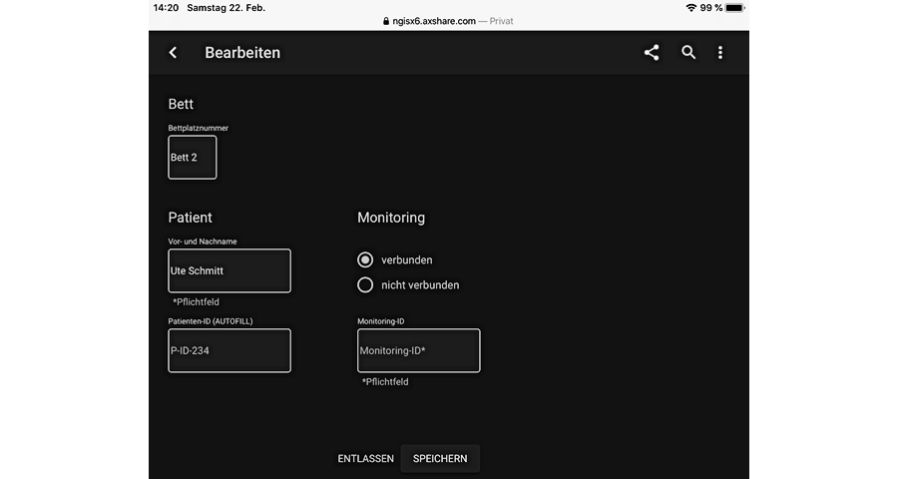
Redesign of the user interface of the prototype (design B) patient admission screen.

**Figure 7:**
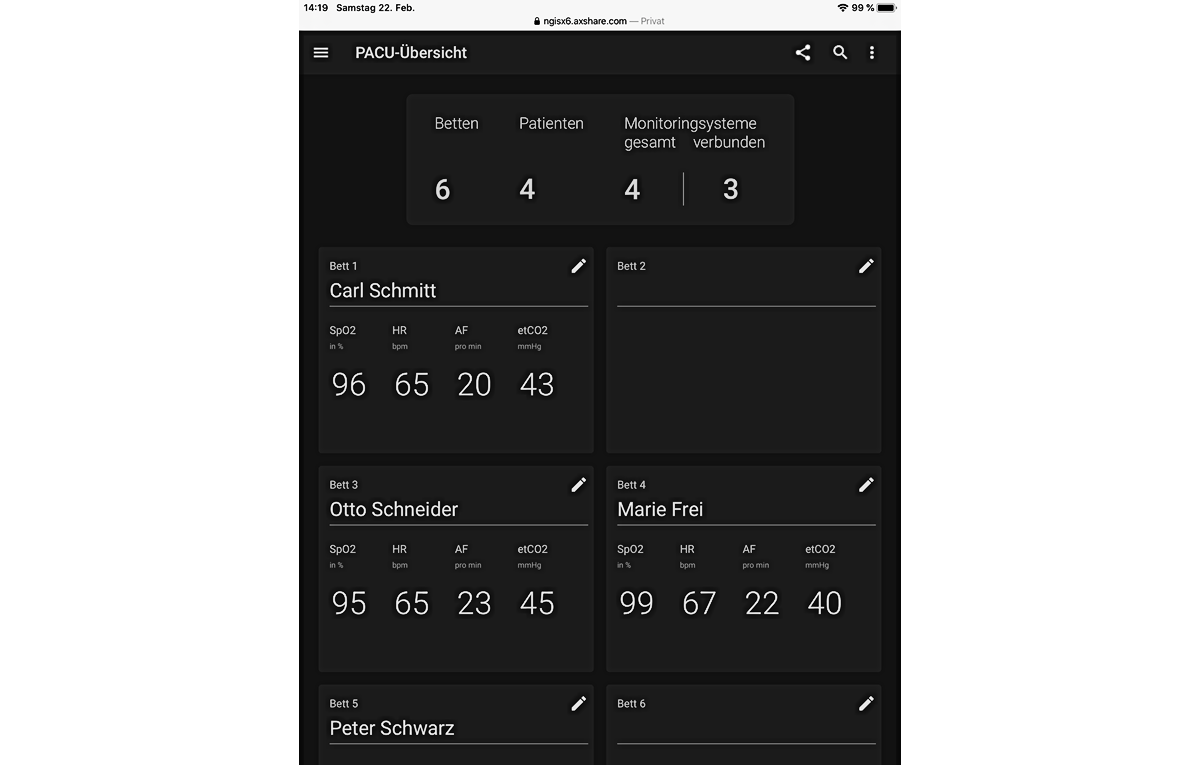
Redesign of the user interface of the prototype (design B) patient tile overview.

The 37 distinct usability problems were ranked in relation to severity and frequency of occurrence (Multimedia Appendix 2). Potential solutions were assigned to the problems and were realized in design B (Figures 6 and 7). In total, 5 design iterations were performed between ASP and LS.

The main improvements in the prototype version compared with the previous interface were as follows:

- More responsive design
- Unknown labels were replaced or removed
- Unknown UI elements were replaced or removed
- A dashboard that counted beds, patients, and monitoring systems was added
- A confirmation dialog before replacing bed numbers was added
- State-of-the-art dark theme design was adapted from material.io

### Quantitative Results

#### Effectiveness

The task completion rate was higher for design B (attempts=20; 0/20, 0% failed and 20/20, 100% passed) than for design A (attempts=18; 7/18, 39% failed and 11/18, 61% passed). A 1-tailed N-1 chi-square test suggests that this is a statistically significant difference (*χ*^2^_1_=9.3; *P*=.002).

#### Efficiency

**Figure 8:**
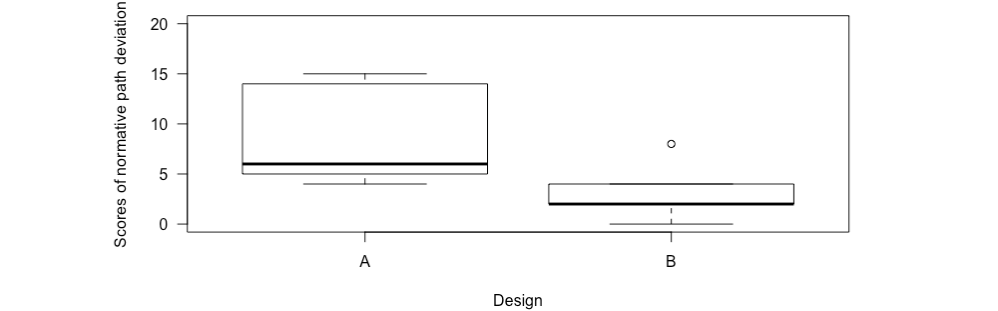
Scores of normative path deviation for design A and design B. The circle symbolizes outliers. Outliers are defined in the box plots as values that have 1.5 times the distance between Q1 and Q3 (Q1 is the lower line of the box, and Q3 is the upper line of the box).

The average normative path deviation of design B (mean 3.2, SD 3.03; 5/10, 50%) was 63.4% lower than that of design A (mean 8.8, SD 5.26; 5/10, 50%; Figure 8). Poisson mixed-effects regression suggests that this reduction in the normative path deviation is statistically significant (*β_design B_*=−1.04, 95% CI −2.09 to −0.13; exp [*β_design B_*]=1.13; *P*<.001).

#### Usability

The average SUS score of design B (mean 89, SD 4.87; 5/10, 50%) was higher than that of design A (mean 68.50, SD 11.26; 5/10, 50%). This difference was statistically significant with a 1-tailed *t* test (*t*_8_=3735; *P*=.003).

## Discussion

### Principal Findings

This study evaluated the usability of a remote patient-monitoring system (design A) by identifying the individual usability problems that informed the conceptualization and design of a revised prototype version (design B). Most of the usability problems identified were related to labeling, followed by the perceived lack of usefulness of the monitoring system [10,53,54]. The UI’s navigation was frequently criticized by participants. Further identified usability problems include unclear UI elements, poor responsiveness, and increased response time. The resolution of the usability problems resulted in a significant increase in the perceived usability, efficiency, and effectiveness of the system.

### Usability of Technologies in Intensive Care Medicine

Over the past 2 decades, the usability of health IT has been investigated in multiple studies applying different methodologies, revealing relatively poor usability and late involvement of end users in the development process [22,55]. This is reflected in our results; based on an HCD approach, we found a relatively high number of easy-to-solve usability problems, the resolution of which led to a significant improvement in the usability of the remote patient-monitoring solution. Most of the usability problems identified were related to labeling, an important issue that is addressed by regulatory requirements [30,56]. The UI’s navigation was frequently criticized by participants. UI navigation problems can affect the overall usability of medical devices, especially in high-stress situations [57-59]. In this regard, simple, intuitive, and role-specific designs are beneficial [60-62], which is also reflected in the user ideas generated by the participants in our study.

The ICU is an exceptional environment that places diverse demands on health IT to be used there. High stress levels and patients who are unstable and critically ill, with varying care and treatment requirements, are among the conditions that must be considered [63-67]. Multiple digital devices already in place increase the cognitive load on staff as they are required to operate the devices and interpret their output [62,67]. Health care professionals applying physiological monitoring systems underuse the range of features currently available [28]. This might also be because of inadequate digital skills among health professionals and insufficient training of staff in the use of digital technologies [68-72].

With the increasing complexity and expanding the functionality of digital technologies and their increased use in all clinical settings, usability considerations have become all the more important to realize the full potential of such innovations. Given our findings, we suggest that HCD plays an important role in realizing the potential of IT in health care.

### HCD in the Implementation of Digital Health Technology

Applying an HCD approach, the inclusion of usability testing and prototyping of a new UI for a remote patient-monitoring system increased usability, according to our findings. HCD encompasses the involvement of end users (ie, health care professionals) in the design and evaluation process, and the required efforts have been shown to be both worthwhile and beneficial in all development phases of a novel digital health technology, enhancing usability and performance [28,59,73]. Research suggests that user knowledge and beliefs about the technology to be implemented are key factors for the successful implementation of the technology [74]. Therefore, HCD should be applied not only during the design and development processes but also during implementation [55]. This could be achieved by establishing innovation and usability laboratories in universities and maximum care hospitals [75]. In the future, HCD is likely to be indispensable for improving both the performance and implementation of IT in health care.

Despite many publications demonstrating the benefits and relevance of usability testing and HCD in health care, there still seems to be a lack of awareness of its importance and the value of involving key users in the early stages of technology development. The reasons for this may be the perceived costs and frequent lack of incentives to conduct usability evaluations. Moreover, as was the case in our study, the design and implementation of health technologies are often separate processes, making it difficult to apply an HCD approach across all development and implementation phases [22,73]. Further research needs to be conducted to explore how to overcome these barriers to obtain the most out of IT products in health care for both staff and patients.

### Limitations

In this study, we showcase an HCD approach to improve the usability of a remote patient-monitoring system in a hospital setting. However, from a scientific perspective, there are several limitations to the scope of the study and the interpretation of results. Owing to the qualitative research design, it is not possible to quantify or generalize the usability problems identified to other health technologies and settings. In addition, translation of our results to other hospital settings or countries is limited because of the single-center design of this study and the relatively small sample size. It was not possible to draw samples randomly, which needs to be considered as a potential source of bias when interpreting the results. The comparison between design A, which was a working medical product installed in the ICU, and design B, a prototype mock-up, may be potentially unfair with a number of confounders in the 2 arms. Nonetheless, given the observed effects of meaningful labeling and easy-to-understand UIs on efficiency and effectiveness, our results help to underline the importance and potential of HCD for realizing the potential of IT in health care. Follow-up studies should be envisioned in collaboration with medical device manufacturers using design B.

We did not perform a usability test of all features of the remote patient-monitoring device, which comprises more than just the remote monitoring device UI (eg, sensors, bedside monitors, or cables are also part of it). We focused on tablet use for this study as it distinguishes remote patient monitoring from regular patient monitoring, and the tablet is the touchpoint with which the user interacts most frequently. Thus, we restricted the study scope to the UI of the tablet version of the remote monitoring system; that is, the smartphone and desktop UI versions were not investigated. We only tested the German version of the UI, which limits certain findings (eg, regarding the labeling) to German-speaking regions.

We were not able to refer to a standardized checklist or protocol for reporting the results of this study. The development of such a checklist or protocol could be an interesting area for further research, as it could improve the quality and reproducibility of usability study reports.

### Conclusions

Applying an HCD approach with usability testing and conceptualized design of a revised prototype version significantly improved the usability of the remote patient-monitoring system for the end points of perceived ease of use, efficiency, and effectiveness. Technical devices should be designed to support efficient and effective work processes, especially in the sensitive working environment of the ICU, with usability being an essential facilitator of maximum performance, successful implementation, and ultimately patient safety. Our results suggest that HCD methods and principles can help realize the goals and potential of IT in health care. However, currently, HCD methods are often not applied early enough in the development process of digital health technologies for ICUs. Further research should explore how to increase early product evaluations in hospitals with end users to take better advantage of their input, not only for the development of user-friendly IT solutions but also for their successful implementation in clinical settings.

## Appendix

Multimedia Appendix 1 Coding scheme adapted from Kushniruk and Patel [44].

Multimedia Appendix 2 Usability ranking.

## Abbreviations

HCD: human-centered design
ICU: intensive care unit
IT: information technology
SUS: System Usability Scale
UI: user interface
