# Supplementary figures and images for: A Remote Patient-Monitoring System for Intensive Care Medicine: Mixed Methods Human-Centered Design and Usability Evaluation
Source: JMIR Hum Factors. 2022 Mar 11;9(1):e30655. doi: 10.2196/30655 (PMC8957007; doi:10.2196/30655)

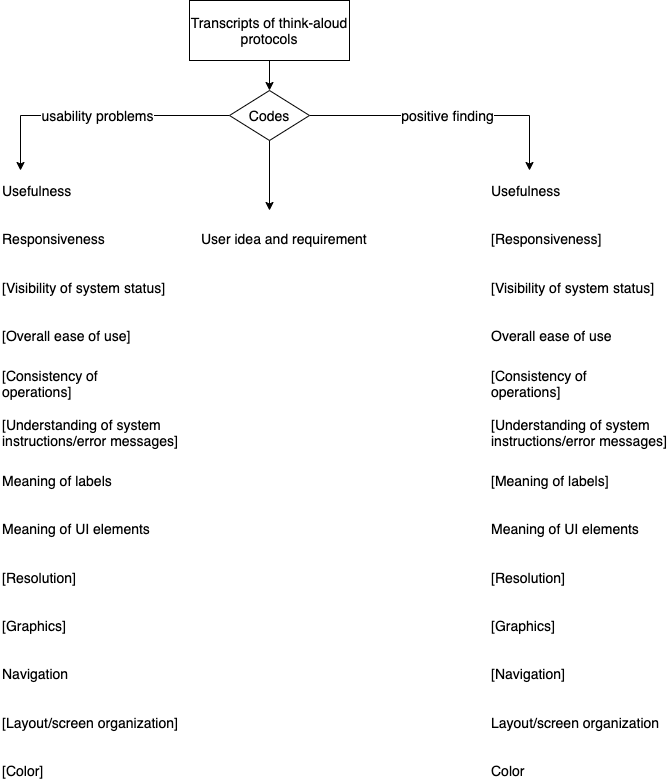

Supplement: Multimedia Appendix 1 [file humanfactors_v9i1e30655_app1.png]
